# Lung Cancer Detection using Probabilistic Neural Network with modified Crow-Search Algorithm

**DOI:** 10.31557/APJCP.2019.20.7.2159

**Published:** 2019

**Authors:** Sannasi Chakravarthy S R, Harikumar Rajaguru

**Affiliations:** *Department of Electronics and Communication Engineering, Bannari Amman Institute of Technology, India.*

**Keywords:** Lung cancer- CT- glcm- chaos theory- crow-search

## Abstract

**Objective:**

Lung cancer is a type of malignancy that occurs most commonly among men and the third most common type of malignancy among women. The timely recognition of lung cancer is necessary for decreasing the effect of death rate worldwide. Since the symptoms of lung cancer are identified only at an advanced stage, it is essential to predict the disease at its earlier stage using any medical imaging techniques. This work aims to propose a classification methodology for lung cancer automatically at the initial stage.

**Methods:**

The work adopts computed tomography (CT) imaging modality of lungs for the examination and probabilistic neural network (PNN) for the classification task. After pre-processing of the input lung images, feature extraction for the work is carried out based on the Gray-Level Co-Occurrence Matrix (GLCM) and chaotic crow search algorithm (CCSA) based feature selection is proposed.

**Results:**

Specificity, Sensitivity, Positive and Negative Predictive Values, Accuracy are the computation metrics used. The results indicate that the CCSA based feature selection effectively provides an accuracy of 90%.

**Conclusion:**

The strategy for the selection of appropriate extracted features is employed to improve the efficiency of classification and the work shows that the PNN with CCSA based feature selection gives an improved classification than without using CCSA for feature selection.

## Introduction

The lung cancer is a disease which can acts as a superior root cause of mortality worldwide among the genders. This type of cancer injures higher amount of lives annually than other cancer diseases like prostate, skin, ovarian and breast diseases (Bray et al., 2018). The lungs are actually bi-spongy structure in the part of chest that assimilates oxygen while inhale and proclaims carbon dioxide while exhale. People (male or female) who smoke will proceed with the ultimate possibility of effect of this cancer and yet this disease can happen for people who don’t smoke too. In these circumstances, the reason of cancer may be not clear. The risk of this disease will rise with the amount of time and amount of cigarette smoked (Hodge et al., 2016). 

Physicians believe that the habit of smoking will result in lung cancer by affecting the lung cells. The cigarette smoke is comprised of toxic agents like carcinogens will affect the lung tissues directly. Thus the inhaling of cigarette smoke influences the cause of lung cancer (Herbst et al., 2018). 

The lung cancer doesn’t make any signs or indications usually in its primary stages (Herbst et al., 2018). The symptoms and indications of this disease normally happen only at its progressive stage. The cure and diagnosis of lung cancer depend on its type, stage and the age of the patients. And the possible actions for curing include radiotherapy or chemotherapy. The endurance of lives of patients is based on the cancer stage, fitness condition and other influences. Clinically there is no certain type of diagnosis method to prevent the lung cancer (Tomasetti and Vogelstein, 2017). On the other hand, the earlier and precipitate discovery of cancer characterizes a significant aspect in the diagnosis and this will upsurge the endurance rate of patients.

The medical phantasmagorias are the substantial way of having treatment for any disease and so for the screening of lung cancer, there is a need for high focus on these medical imaging techniques. These medical photograph contain out of sight facts and features which will be applied by the clinical specialists to take appropriate verdicts for the diagnosis of the patients. Thus the extracting and utilizing these appropriate hidden facts diagnostically becomes the first step. This in need make use of any data-mining methods for the extraction of information from clinical images. This involves the pre-processing of input lung images used to have an improved lung image quality for the auxiliary manner of extracting the features. 

The CT lung dicom (.dcm) medical input images are utilized for this study and they are taken from the database of Lung Image Database Consortium (LIDC). This image dataset is composed of both diagnostic and cancer screening thoracic CAT examinations along with marked-up interpretations (Armato et al., 2011). The LIDC is an easily accessible in internet which forms a global reserve for expansion, training and assessment of computer-assisted diagnostic (CAD) approaches for the discovery or finding the cancer disease and for further treatment. This is genuinely originated by the National Cancer Institute and additionally progressed further by the Foundation for the National Institutes of Health (FNIH) which is altogether supported by the Food and Drug Administration (FDA) through active involvement. This kind of good partnership between public and private makes evident about the achievement of an association initiated on an agreement-based method (McNitt-Gray et al., 2007). The academic centres of about 7 and medical imaging enterprises of about 8 have teamed up to construct this database with 1,018 different studies. In this, every case contains examinations through medical thoracic CT photograph with related eXtensible-Markup Language file to note down the fallouts of a bi-phase image mark-up approach done by four skilled radiologists (Reeves et al., 2007).

**Figure 1:**
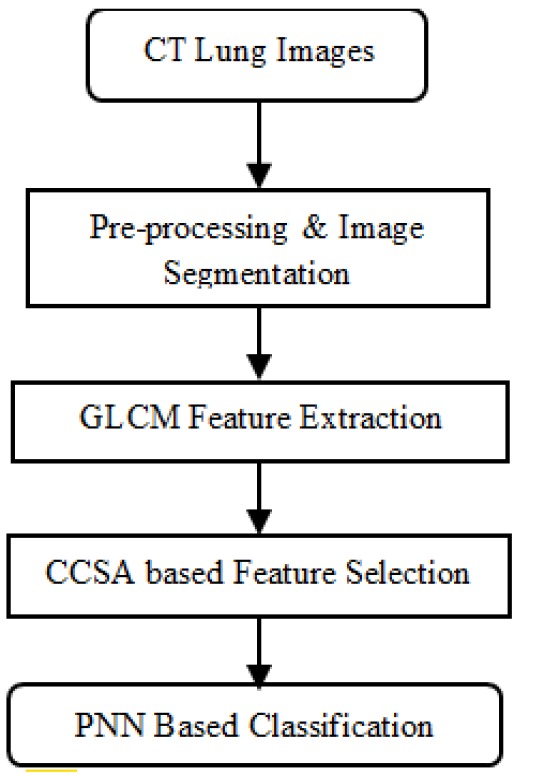
Proposed Method

The CT slices used in this paper have a 16-bits intensity with 512 × 512 image resolution, the slice thickness ranged between 0.5 to 2.5 mm and the overall slice number for every individual scan ranged with an average value of 136/scan. The intention of this paper is to provide an instinctive classification of lung cancer for its earlier and precipitate discovery and diagnosis. For this theme of classification, the probabilistic neural network is used. Then Gray-Level Co-Occurrence Matrix way of feature extraction and CCSA Algorithm based selection of significant features is done on obtained texture features for better results. This methodology is shown in the Figure 1.

## Materials and Methods

*A. Pre-processing and Segmentation*

The pre-processing of input CT images is essential to diminish the noise in it and make the input images suitable for ensuing paces like image segmentation. This will reduces distortions in input images and so augments the appropriate features of inputs. MATLAB 2013a software is used for pre-processing of input CT images.

**Figure 2:**
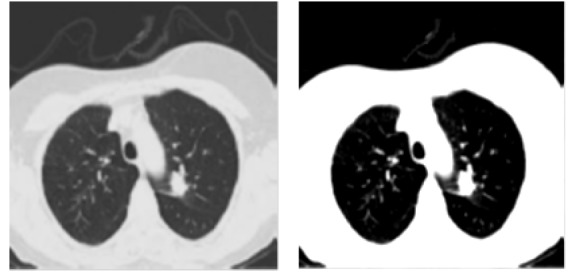
Cancerous and Binary CT Lung Image

In the input database, the study considers both primary and secondary phase cancer nodules with 4 distinct nodule types such as Well-circumscribed type, Juxta-pleural type, Vascularized type and Pleural-tail type nodules. The Figure 2 shows the input CT lung image with cancer.

The lung CT images are filtered using fast-adaptive median type of filter (Chakravarthy and Subhasakthe, 2015) for removing the impulse noise by maintaining the fine information in it. The lung portion will be separately segmented from the input lung CT images through morphological process. At first, the input of type gray-scale lung image is transformed to binary image type. This is simply done by making the entire pixels in the input CT lung image having intensity value larger than the taken threshold value is then supplanted with ‘1’. Similarly the pixels having intensity value smaller than the taken threshold value is then supplanted with ‘0’. The value of threshold for this conversion is computed by mixture modelling based automatic thresholding method (Abirami et al., 2016). This thresholding method selects the value of threshold such that reducing the intra-class variance of the binary-valued pixels and this is shown in Figure 2.

**Figure 3:**
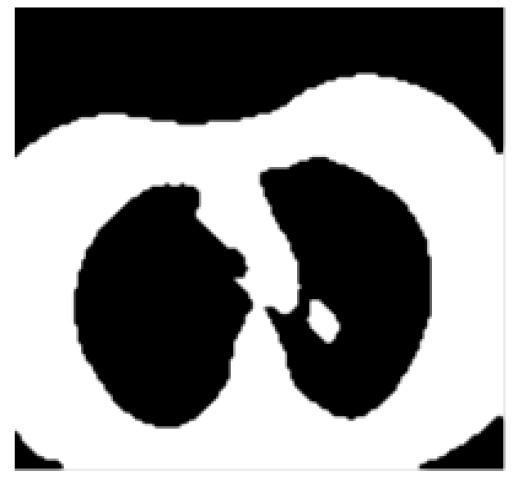
Morphological Output

The morphological operation of opening is done to the binary converted CT lung input image with a structuring element which refers to shape, has to interact with a taken input image. In general, a structuring element refers to a matrix which ascertains the image pixels being deal with and outlines the neighbourhood utilized in the processing of respective pixel (Haralick et al., 1987). The purpose of this is to draw the conclusions in what way this considered shape fills or slips with the shapes in the input CT lung image. For this the morphological structuring component chosen is ‘periodic line’ that refers to a flat element with 2 × (L + 1) members. The notation ‘L’ denotes the size of the morphological structuring component and the value of L is chosen as two in this study. The middle pixel of the structuring component is termed as the origin which is used for finding the pixel being deal with. The Figure 3 represents the morphological output image.

**Figure 4:**
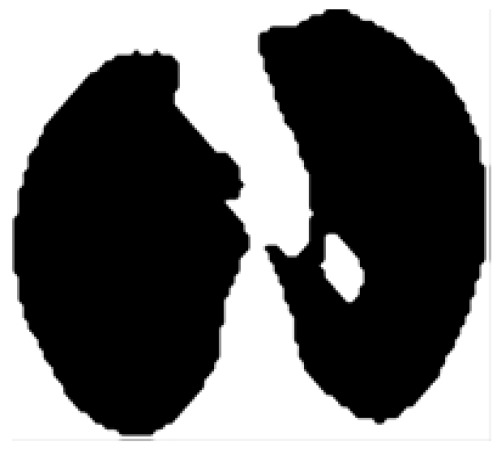
Output after Segmentation

The image of Figure 4 is now reversed and then the operation of achieving clearing-border process is carried out. This is used to clear the structures which are lighter than its neighbours interconnected to the border of the segmented image (Ronneberger et al., 2015). The Figure 4 denotes final output obtained after the segmentation process.

*B. Feature Extraction using GLCM*

After the input CT lung images have been segmented for its region of interest, the extraction of GLCM features is done. Feature extraction is the process of reformation of larger raw input image data into a pack of features (Abirami et al., 2016). These features will have unique properties of taken patterns and gives an easier way in discriminating among the sets of input image patterns. It is simply a technique to denote the considered input image in an abridged data and so this provides an easier way of interpretations in classification, detection or recognition problems. Hence it will reduce the volume of raw images by refining its descriptive means of attributes. The feature extraction at all times commences from a primary set of raw image and then constructs the resultant derived features which is to be more useful but need to be non-redundant (Abirami et al., 2016).

The texture analysis based on statistical properties analyses the region of interest through the texture in an image by means of their higher-order indices of grayscale histograms. And the most popular texture analysis tool is GLCM (extracting textural attributes from Gray-Level Co-occurrence Matrix). This texture analysis method depends on the second-order statistics of computed histograms of pre-processed and segmented images (Mohanaiah et al., 2013). Haralick and Shanmugam, (1973), proposed the co-occurrence matrix and texture based attributes. He introduced two ways for the extraction of texture features: 

i. The primary step is determining the co-occurrence matrix,

ii. Next phase is computing the texture attributes according to the former determined co-occurrence matrix.

The feature extraction using GLCM is found to be a good choice for many analysis ranging from medical imaging to remote sensing applications (Kuffer et al., 2016). The GLCM defines how frequently a pixel with gray-scale intensity i take place horizontally in line to a pixel having the value j. The offset parameter in glcm is a matrix that will controls the relative spacing of pixels to check so as to obtain the co-occurrence total of individual value pair. The offset value taken in the work is [2 0; 0 2]. The first matrix row is [2 0] which indicates that every individual pixel is related to the pixel of two-rows down with zero columns (same column) over. And the second matrix row [0 2] means that every individual picture element is also related to the picture element zero rows (same row) away with two-columns over. Accordingly every individual picture element is compared in contrast to two of its adjacent picture elements; the corresponding picture element is two-columns to the right and two-rows down. Thus the pairs generated by these relationships will be utilized in order to increment the respective picture elements in the taken image. The computed thirteen features for a sample CT lung image (ID is LIDC-IDRI-0003 with 4 nodules greater than or equal to 3mm) in the LIDC dataset is given in Table 1.

As shown in Table 1, the features acquired through GLCM is huge and it will be reduced by using chaotic crow search algorithm (CCSA), which is detailed in the following subsection.

*C. Feature Selection using CCS Algorithm*

Feature or attribute selection is primary type of core perception in machine learning such that it will incredibly influences the performance of the chosen classifier. The input features which are used to train the classifier models will have a vast impact on its performance. It is the method of manually or automatically choosing the features from the extracted features. Devising inappropriate features in the input can drop the accuracy of the classifier and will leads to wrong decision. The process of attribute selection can simply aid as a filter which muting out the attributes that won’t be beneficial besides to the extracted features (Li et al., 2018).

In 2016, Crow search algorithm (CSA) is a natural motivated method introduced by (Askarzadeh, 2016) for optimization problems. The concept behind CSA is naturally based on the crow-search technique for their food hiding manner. Similar to other optimization methods, the CSA has the limitation of lower convergence ratio. The chaos is a kind of mathematical methodology used to improve the performance of evolutionary algorithms like CSA. The paper uses chaotic crow search algorithm (CCSA) to overcome the convergence rate limitation of CSA. The CCSA is implemented to select the GLCM features. The sine chaotic map is employed for the optimization process of CSA. This implies that a chaotic-binary model of CCSA is implemented to improve the result of CSA.

In CCSA methodology, the chaotic search approach is employed to choose the optimal feature subset that improves reduces the length of feature subset and increases the output accuracy (Sayed et al., 2019). The sine chaotic map is taken and substituted with arbitrary movement variables of CSA. In CSA, the arbitrary parameters utilized for updating the position of crow are substituted with chaotic parameters. This will yields optimal solution with better convergence rate. 

The sine chaotic map (Fridrich, 1998) with range (0, 1) is defined as

$p_{q+1}=\frac{c}{4}\mathrm{Sin}(\pi p_{q})$

(1)

where *q* in above equation (1) represents the index of sine chaotic map sequence *p*, the *q*^th^ number in sine chaotic sequence is denoted by *p*_q_ and *c* is the control parameter with value of 4 used to find the chaotic behaviour of the model. The starting *p*_0_ point is taken as 0.7 for the sine chaotic map; this chosen initial value should have a huge impact of fluctuation parameters on sine chaotic map. The range of above equation (1) is (0, 1) denotes that it is a binary CCSA approach. In this approach, solution pool is in the kind of 0 and 1, indicating that the solutions are constrained to (0, 1).

The CSA method adjoined with chaotic sequence (Kohli and Arora, 2018) is defined as


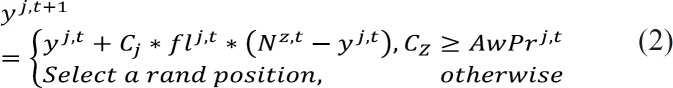


where *C(j)* denotes the attained value of sine chaotic map at *j*^th^ iteration, *y*^j,t^ is the current position, *y *^j,t+1^ is the updated position, *fl *is the flight length, N is the best obtained position, *C(z)* indicates the attained value of sine chaotic map at *z*^th^ iteration and *AwPr*^j,t^ is the parameter of awareness probability at *t* iteration of crow *z. AwPr* is responsible for the maintaining the steadiness between exploration and exploitation. If its value is low, then it will result in the search on local regions and this is referred as exploitation. If its value is high, then it will result in the global search on search regions and this is referred as exploration. The initial parameters of CCSA are considered as number of crows *M* as 25, *AwPr* as 0.1, *fl* as 2 with maximum number of iterations* t*_max_ as 50. Thus the CCSA algorithm (Sayed et al., 2019) is given as

Set the values of initial parameters.

Initialize the position of crow y arbitrarily.

Compute the fitness function of individual crow.

Start the initialization of the memory of search crow N.

Start with t=1 (initial iteration).

repeat

for (*j*=1:M) do

Obtain the c value in sine chaotic map

If *C*_Z_≤ *AwPr*^z,t^

*y* ^j,t+1^ *= y* ^j,t^ *+ C*_j_ * fl ^j,t^ * (N ^z,t^ *-y* ^j,t^ *)*

else

*y* ^j,t+1^ *=A (*any random position*)*

end if


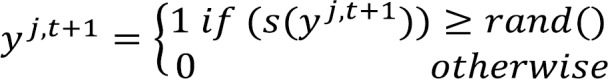


end

Examine the possibility of *y *^j,t+1^

Now update the memory of crow

Increment the iteration *t=t+1* up to *t<t*_max_*.*

Reduced feature subset is obtained.

Out of thirteen extracted features using GLCM as in Table 1, the CCSA selects only six significant features: Angular Second Moment, Contrast, Correlation, Variance, Entropy and Homogeneity. These six reduced features are considered as primary and other seven features are secondary which are derived from the primary features. After attaining the significant and condensed feature set, they are all set to deliver as input to the PNN classifier. The extraction, selection of features and classification of the processed input CT lung images are done using MATLAB R2013a.

*D. Classification using PNN*

A probabilistic neural network (PNN) was introduced in 1990 by Donald Specht (Specht, 1990), is a tri-layer feed-forward architecture with one-pass training methodology utilized for the applications of data mapping and data classification. The core of PNN is based on the popular statistical principles resulting from Bayes decision theory and non-parametric kernal estimators based probability density functions (pdf). The core function of PNN is to compute the pdf of features of every individual class provided from the input training sample data with Gaussian Kernel. And the computed pdf are then utilized in Bayes decision theory to carry out the data classification (Morin and Bengio, 2005). 

PNN integrates the neural network with statistical theory concept that leads to an influential approach for classification task where the conventional statistical equivalents become unsuccessful. A PNN is basically a Bayes–Parzen type classifier. The PNN delivers a universal solution to the classification problems by using a technique introduced in statistics named Bayesian classifiers. This network has a primary advantage of its training speed is faster than a back-propagation neural network and it is easier to train the inputs (Wu et al., 2007). In this, it is assured to tactic the Bayes optimal decision surface on condition to that the class pdf are smooth and continuous. 

**Figure 5:**
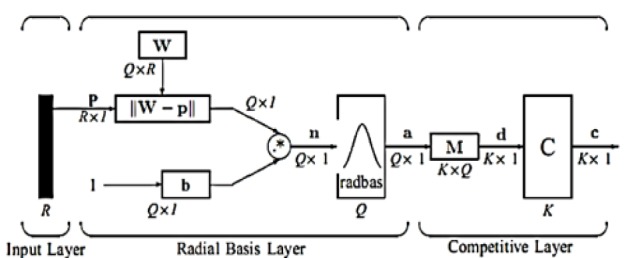
Structure of PNN

**Figure 6:**
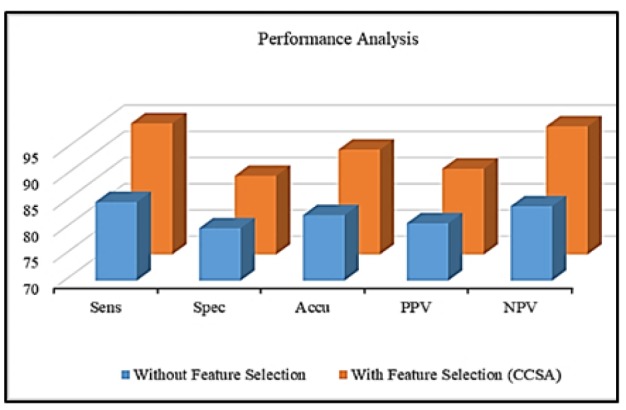
Graphical Analysis

**Table 1:** Sample Extracted GLCM Features from an Input CT Lung Image

S.No	Features	Value
1	Angular Second Moment	[1.312141414744435e+00 1.311621145792002e+00]
2	Contrast	[7.015931372549020e-03 7.184436274509804e-03]
3	Correlation	[9.788619561710441e-01 9.783478994727116e-01]
4	Variance	[-7.575756470094511e-01 -7.572759361235961e-01]
5	Inverse Difference Moment	[7.824877982000489e-01 7.820374071686120e-01]
6	Sum Average	[9.579810049019606e+00 9.579978553921569e+00]
7	Sum Variance	[8.220227931311725e+01 8.219470229585625e+01]
8	Sum Entropy	[5.495567337778960e-01 5.501246317841957e-01]
9	Entropy	[5.544198068277803e-01 5.551045035317845e-01]
10	Difference Variance	[7.015931372549020e-03 7.184436274509804e-03]
11	Difference Entropy	[4.178727739018122e-02 4.261978134521981e-02]
12	Information Measures of Correlation	[7.015931372549020e-03 7.184436274509804e-03]
13	Homogeneity	[9.964920343137255e-01 9.964077818627450e-01]

**Table 2:** Standard Performance Metrics Used for Evaluating the Proposed Model

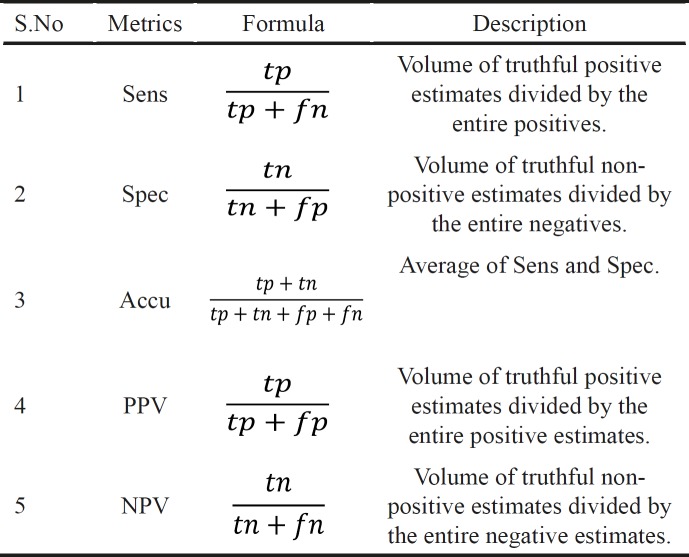

**Table 3:** Training and Testing MSE of PNN Classifier

Particulars	Features	Average Training MSE	Average Testing MSE
Method I	All 13 GLCM Features	1.62 x 10^-4^	1.31 x 10^-4^
Method II	Reduced GLCM Features using CCSA	1.27 x 10^-5^	1.18 x 10^-5^

**Table 4:** Performance Comparison of the Proposed Model

Metrics (%) →	Sens	Spec	Accu	PPV	NPV
All 13 GLCM Features	85	80	82.5	80.95	84.21
Reduced Features by CCSA	95	85	90	86.36	94.44
Improvement	10	5	7.5	5.41	10.23

**Table 5:** Comparison of Computation Time of Proposed

Particulars	GLCM Feature Extraction (MATLAB)	CCSA Algorithm (MATLAB)	PNN (MATLAB)	Total Time in seconds
	in seconds	in seconds	in seconds	
Method I	32.4	-	238	270.4
Method II	32.4	59.2	44.7	136.3

In PNN, the weights used in the network are not “*trained*” however assigned and the prevailing weights won’t be changed on the other hand merely new vectors are introduced into weight matrices during the process of training. This will make PNN to use in real-time applications. Subsequently the speed of the network is very fast because the running and training procedure can be carried out by matrix manipulation. The PNN can classify the input features into a particular class since that obtained class will have a higher probability to be accurate (Khan et al., 2015).

The structure of PNN architecture is given in Figure 5. PNN has three layers: input, radial basis and competitive layer. The radial basis layer is used to compute vector distances among the input and row-weight vectors. These vectors are found in the weight matrix. The computed vector distances are then scaled non-linearly by radial basis function. Now the third, competitive layer estimates the shortest distance among these vectors. Thus determines the training attribute approximates to the input attribute according to their distance array (Othman and Basri, 2011). The symbols and descriptions are taken as given in the mathworks toolbox (neural network).

In the input layer, the input vector is represented as **p** with R × 1 dimension which is given as a dark vertical line in Figure 5. The work considers that the value of R is 3. The vector distances among input vector **(p)** and the row-weight vector of weight matrix **W** of radial basis layer are estimated. Consider the dimension of weight matrix is Q X R and this vector distance calculation represents dot-product among the two vectors. As shown in Figure 5, the dot-product concerning **p** and *i*^th^ row of **W** gives distance vector **||W-p||** with the dimension of QX1. The symbol (-) in distance vector denotes the distance between those distance array. At that moment, with this obtained distance vector, the bias vector **(b)** is included by means of element-by-element multiplication (.*). As a result, **n = ||W-p||** .* b is obtained. The PNN transfer function used in the work is defined as:

 (3) $$\mathrm{radbas}\left(n\right)=e^{{-n}^{2}}$$

In the above equation (1), each element of obtained n value is replaced for generating the output vector of radial basis layer **a**. The* i*^th^ element of **a** is denoted as 

$a_{i}=\mathrm{radbas}(∥w_{i}-p∥∙*b_{i})$

 (4)

where *W*_i_ represents the vector of *i*^th^ row of weight matrix **W**, *b*_i_ indicates the *i*^th^ element of **b** and .* denotes the element-by-element multiplication. A radial basis neuron having a weight-vector near enough as the input vector **p** will gives an approximated output as **1**. This output weights will pass their vectors to the next competitive layer. 

In competitive layer, there is an absence of bias and the radial basis layer output vector **a** is going to have a product with the layer weight matrix **M** to generate an output vector **d**. Now as in Figure 5, the output corresponds to competitive function (C) will generates a **1** related to the biggest element of **d**, otherwise produces **0**. The resultant vector of competitive layer is denoted by **c**. The output of **1** in competitive layer c gives the amount of tumor that the considered model can classify. 

## Results

The enactment of the study proposed is valued by benchmark metrics: Sensitivity (Sens), Specificity (Spec), Accuracy (Accu), Precision (PPV) and Negative Predictive Value (NPV) (Glas et al., 2003). The description of these metrics and how their values are estimated are given in Table 2. And they are valued using confusion matrix which includes true and false positive and true and false negative. The true negative and positive envisage that the cases are diseased and non-diseased in which they are in fact diseased and non-diseased. The false negative and positive are simply contradictory to the true negative and positive (Veropoulos et al., 1999). These are known by the notations tp, tn, fp and fn.

The proposed model uses standard 10-fold testing and training (Abirami et al., 2016), where 90% and 10% of input features are used for training and testing. The Mean Square Error (MSE) is defined as (Abirami et al., 2016) 

(5) $$\mathrm{MSE}=\frac{1}{N}\sum_{i=1}^{N}{\left(T_{i}-O_{i}\right)}^{2}$$

where T and O indicate the target and obtained result during the classification using PNN. The N denotes the number of input CT lung images. The average MSE value for training and testing the PNN is shown in Table 3. The reduced GLCM features using CCSA with PNN produces a better value of MSE which makes this method to give better accuracy as in Table 4. 

The performance comparison of the proposed model is depicted in Table 4. The Table 4 shows that all the benchmark metrics are settled high for the proposed system that uses CCSA based feature selection. It is also observed that overall 7.6% improvement of all metrics is achieved when compared with the entire GLCM features using PNN classifier. The comparison is graphically illustrated in Figure 6.

Computation time is the amount of time taken by the system to obtain the required results. The work uses Intel Core i3 II generation processor based 4 GB RAM computer with MATLAB 2013a environment. The obtained computation times on running the corresponding program for both the techniques are given in Table 5 where method I denotes the classification using PNN with entire GLCM features and method II represents the classification by PNN with reduced GLCM features using CCSA. It is seen that the computation time of method II is almost half of method I.

The robustness of PNN and higher convergence property of CCSA towards the output classes make the proposed model to perform well better in both the aspect of accuracy and the time taken for computation.

The comparison with one lung cancer CAD scheme and another methodology is a hard one because various aspects like input database, way of extracting the features, method of selecting the appropriate features and type of classifier can have an impact on classification accuracy of the proposed one. However, the result of the proposed model is compared with other related works that adopt various feature extraction methods with different classification algorithms in Table 6. And in all the works, lung CT images were used to examine the performance. From Table 6, the proposed model works well than the existing schemes. The presence of outliers in GLCM features will limit the performance of the proposed classifier. The extraction of wavelet features, morphological features and various entropies can remove the limitation of GLCM features.

## Discussion

The classification of lung cancer using GLCM features for CT images is discussed in this paper. These features are given as input to the PNN classifier and attained the classification accuracy of 82.5%. In order to improve the classification accuracy, a strategy of selecting appropriate features with CCSA algorithm is employed in this paper. This leads to the improvement of classification accuracy of 7.5% when compared with the performance of PNN with entire GLCM features as input. Further research is in the direction of selecting statistical, geometrical and Gabor features and with the inclusion of deep learning strategies.
